# Clinical and financial impacts of abnormal liver biochemistry after liver transplantation

**DOI:** 10.1186/s13104-022-06268-w

**Published:** 2023-01-27

**Authors:** Dee Zhen Lim, Nicholas Low, Louise Jackett, Ronald Ma, Robert Jones, Adam Testro, Laurence Weinberg, Muralidharan Vijayaragavan

**Affiliations:** 1 Department of Surgery, Austin Health, 145 Studley Road, Heidelberg, VIC 3084 Australia; 2 Department of Pathology, Austin Health, Heidelberg, VIC Australia; 3 Department of Finance, Austin Health, Heidelberg, VIC Australia; 4 Department of Gastroenterology, Austin Health, Heidelberg, VIC Australia; 5 Department of Anesthesia, Austin Health, Heidelberg, VIC Australia

**Keywords:** Liver transplantation, Health economics, Liver function tests

## Abstract

**Objective:**

After liver transplant (LT)**,** many investigations are needed to evaluate abnormal liver function test (LFT), which has poor specificity for graft function and complication. A single center retrospective audit of all adult single organ LT from 1/1/2015 to 31/12/2017 was performed. Demographic, clinical and investigation data from the LT database and electronic medical records and cost data from the hospital’s Business Intelligence Unit were analyzed. Patients were classified into uncomplicated or complicated LFT by 2 independent investigators and the number, type, and cost of investigations in the first 30 post-operative days were analyzed. Investigations prior to liver biopsy was sub-analyzed.

**Results:**

There was 170 LT with 87 cases of uncomplicated LFT (51.2%) and 83 cases of complicated LFT (48.8%). Most patients with complicated LFT had additional investigations (97.6%), most commonly cholangiogram (55.4%) and liver biopsy (LBx) (50.6%). The additional investigations cost was $1863.3 (95% CI 1289.0–2437.6). Although most LBx (73.8%) showed evidence of rejection, LBx was often not the initial investigation of choice. Current LFT based post-transplant monitoring is inefficient. It remains difficult to determine which patient will benefit from an early invasive procedure like LBx, using LFT alone without further imaging investigations.

**Supplementary Information:**

The online version contains supplementary material available at 10.1186/s13104-022-06268-w.

## Introduction

Liver function tests (LFTs) are the cornerstone of post liver transplant monitoring. However, while LFTs are highly sensitive, they are a poorly specific metric for assessing postoperative graft function and complications [1, 2]. LFT derangement post transplant is traditionally investigated with various imaging modalities, including ultrasound (US), computer tomography (CT) or MRI (magnetic resonance imaging), as well as more invasive modalities such as endoscopic retrograde cholangio-pancreatography (ERCP), percutaneous cholangiography or percutaneous liver biopsy (LBx). This escalating pattern of numerous imaging modalities being undertaken to diagnose graft function frequently results in a significant delay of a definitive diagnosis and subsequent therapeutic intervention. In an era of fiscally constrained healthcare resources and emergent low-cost biomarkers, defining the patterns of investigations following LFTs derangement, and their associated economic costs, is an urgent requirement for efficient and cost-effective management of LT and its complications.

## Main text

### Methods

We conducted a single center (Austin Health, Melbourne, Australia) retrospective audit of consecutive adult patients (age greater 18 years) undergoing single-organ liver transplantation (LT) between 1 January 2015 and 31 December 2017. Multi-visceral transplants, combined liver-kidney transplants, and redo LT within 30 days of the index transplant were excluded because LFTs in this setting are not comparable. The study was approved by Austin Health Human Research Ethics Committee (LNR/18/Austin/107) and a waiver of participant consent was granted. The primary objective was to determine the number, type, and cost of additional investigations for LFTs derangements in the first 30 post-operative days.

Baseline patient variables, clinical indications, operative details, and results of investigations were collected from the LT unit database and the hospital’s electronic medical records. Additional investigations were prespecified and included ultrasound abdomen (US), US doppler abdomen 4 days after LT, computed tomography abdomen pelvis (CTAP), magnetic retrograde cholangio-pancreatography (MRCP), endoscopic retrograde cholangio-pancreatography (ERCP), liver biopsy (LBx) and cholangiogram. The costs in Australian dollars of additional investigations were collected from the hospital’s Business Intelligence Unit. The cost of LBx comprised the procedural costs as well as the cost of histology examination and immunochemistry staining. The cost of ERCP comprised the procedural cost and associated operating theatre times and expenses.

Patients were classified into uncomplicated or complicated LFTs by 2 independent investigators, who were blinded to the results of other investigations. Uncomplicated LFTs was defined as an LFT that followed the expected postoperative course. This included serum alanine transaminases (ALT) reaching a peak approximately 2 days after LT and trending down thereafter, and serum alkaline phosphatase (ALP) and gamma-glutamyltransferase (GGT) peaking approximately 7 to 10 days after LT and trending down thereafter (see Additional file 1: Figure S1A) [3]. Complicated LFTs was defined as an LFT that did not follow the expected postoperative course (see Additional file 1: Figure S1B). Rejection was graded using the Rejection Activity Index (RAI). Any differences were then reconciled between the two investigators. The number, type, and cost of additional investigations between the uncomplicated versus the complicated LFT group were analyzed. Additional investigations prior to LBx were sub-analyzed.

### Statistical analysis

Continuous variables were expressed as means and standard deviations. Categorical variables were expressed as counts and percentages. One-way analyses of variance and independent Student’s *t* tests were used to compare continuous variables, while Pearson’s chi-squared test or Fisher’s exact test were used to compare categorical variables. The study was reported according to The Strengthening the Reporting of Observational Studies in Epidemiology (STROBE) guidelines [4].

## Results

170 LTs were analyzed, of which 87 (51.2%) had uncomplicated LFTs and 83 (48.8%) had complicated LFTs (see Additional file 1: Figure S2). There were no statistically significant differences observed in the baseline characteristics of patients with uncomplicated versus complicated LFTs (see Additional file 1: Table S1).

The number of investigations performed was higher in patients with a complicated LFTs than in patients with an uncomplicated LFTs (see Additional file 1: Figure S3). There was a significant correlation between complicated LFTs and number of investigations, p-value < 0.001. Fifty-four percent of patients with an uncomplicated LFTs had no additional investigations, 24.1% had one postoperative investigation, 14.9% had two and 6.9% of patients had three additional investigations. In comparison, 97.6% of patients with a complicated LFTs had additional investigations.

**Fig. 1:**
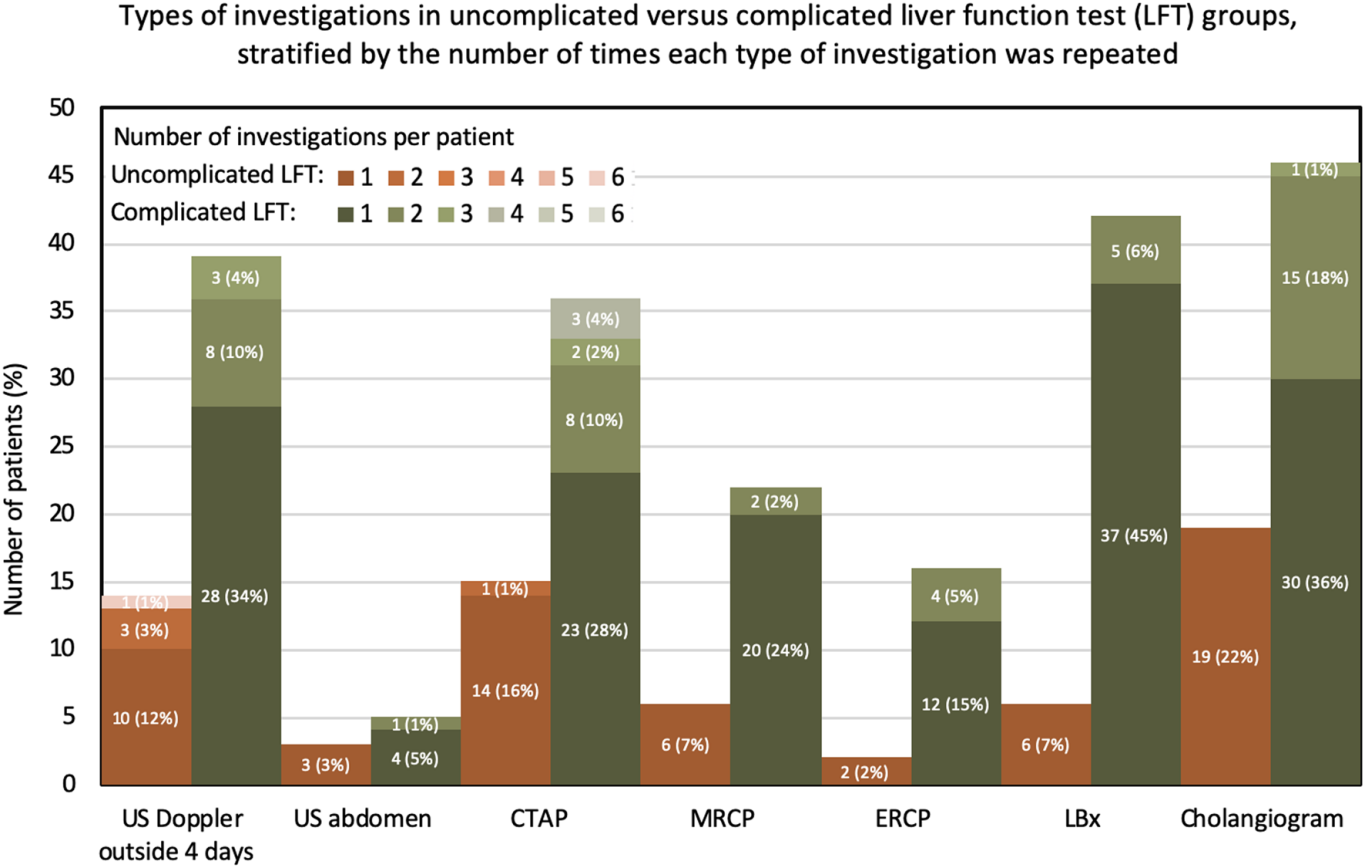
Comparison of the types of investigations in uncomplicated versus complicated liver function test (LFT) groups, stratified by the number of times each type of investigation was repeated. *US* ultrasound, *CTAP* computed tomography abdomen pelvis, *MRCP* magnetic resonance cholangio-pancreatography, *ERCP* endoscopic retrograde cholangio-pancreatography, *LBx* liver biopsy

The types of additional investigations performed are shown in Fig. 1. There was a significant association between patients with complicated LFTs and multiple repeated investigations of the same imaging modality (p-values < 0.01). The three most common additional investigations in the complicated LFT group were cholangiogram (55.4%), LBx (50.6%) and US Doppler beyond 4 days (47.0%). All cholangiograms performed were tube cholangiograms except one.

### Liver biopsy sub-analysis

In this group, 53 LBxes were performed in 48 patients. Of these, 43 patients had one LBx, while five had two procedures. The majority of LBxes occurred in patients with a complicated LFT (*n* = 42, 87.5%); patients with an uncomplicated LFT rarely had LBxes (*n* = 6). All five patients who had two LBxes belonged to the complicated LFT group. The median time to LBx was 9 days (interquartile range = 7–14).

**Fig. 2:**
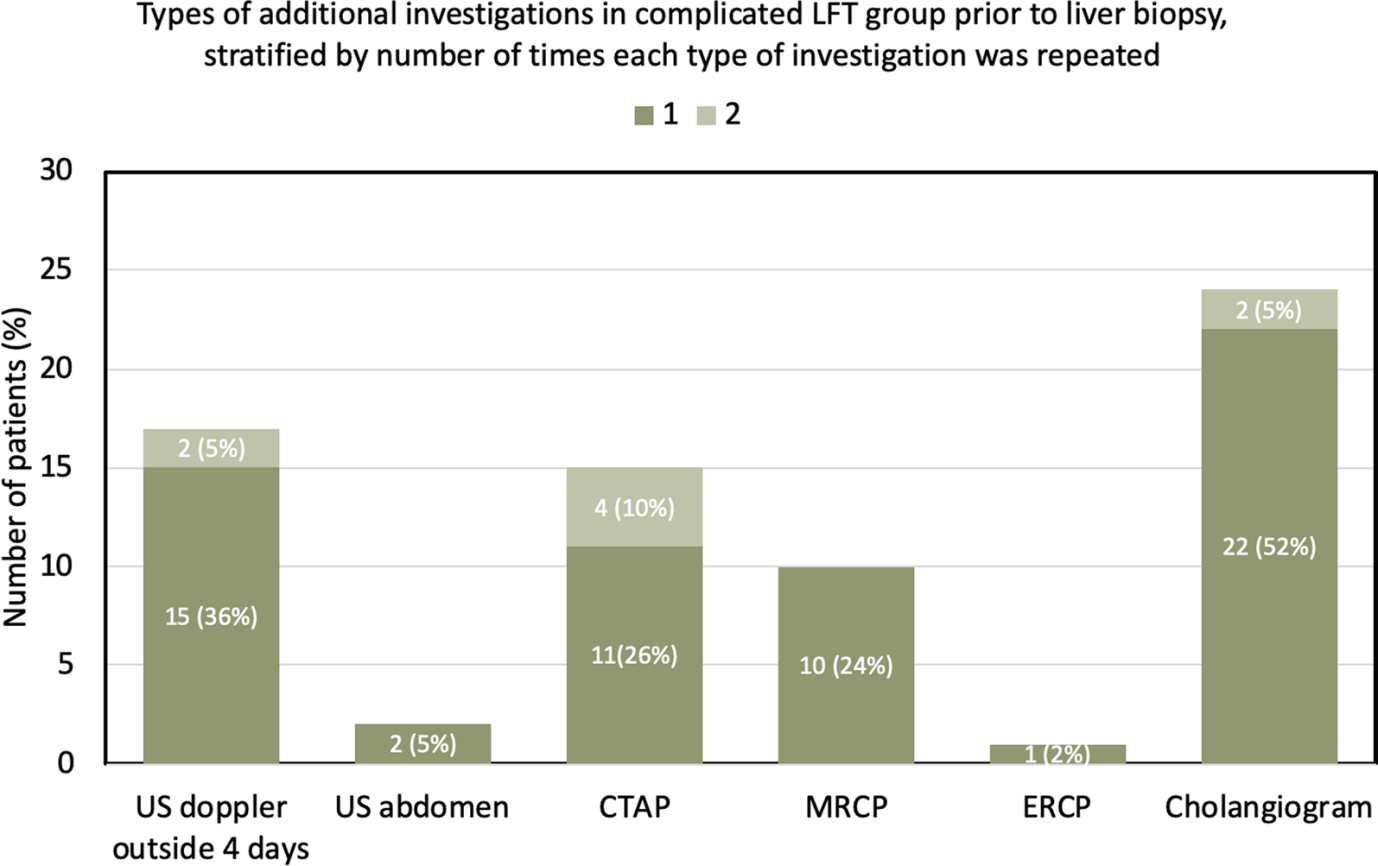
Types of additional investigations in the complicated liver function test (LFT) group prior to liver biopsy, stratified by the number of times each type of investigation was repeated. *US* ultrasound, *CTAP* computed tomography abdomen pelvis, *MRCP* magnetic resonance cholangio-pancreatography, *ERCP* endoscopic retrograde cholangio-pancreatography

The LBxes of all patients who had a complicated LFT were preceded by additional investigations. Approximately half had one additional investigation (*n* = 19, 45.2%) while the rest had two (*n* = 15, 35.7%), three (*n* = 5, 11.9%) or four additional investigations (*n* = 3, 7.1%). The type of additional investigation performed for patients with complicated LFTs prior to LBx is shown in Fig. 2. The three most common investigations were tube cholangiogram (57.1%), US Doppler beyond 4 days (40.5%) and CTAP (35.7%).

The majority of the LBx demonstrated evidence of rejection. Of the patients who had a LBx in the uncomplicated LFT group (*n* = 6), five had biopsy-proven rejections — one variant cellular rejection, two mild rejections (RAI 3–4), one moderate rejection (RAI 5–6) and one severe rejection (RAI 7–8)—and the LBx of one patient showed no rejection, only ischemia–reperfusion injury. The clinical characteristic and LFT profile of these six patients are presented in Additional file 1: Figure S4. Of the patients who had LBx in the complicated LFT group (*n* = 42), 31 had biopsy-proven rejections: one humoral rejection, 12 mild rejections (RAI 3–4), 13 moderate rejections (RAI 5–6) and five severe rejections (RAI 7–9). The remaining 11 patients in this group showed no evidence of rejection. For patients who did not have a biopsy-proven rejection in the complicated LFT group (*n* = 52), the cause of LFT derangement was identified as vascular-related (*n* = 3), biliary-related (*n* = 10), sepsis (*n* = 8), graft-related (*n* = 3), ischemia–reperfusion (*n* = 5) and small bowel obstruction (*n* = 2). Several patients had multiple reasons for LFT derangement within the first 30 postoperative days (*n* = 2). A definitive cause was not identified in the remaining 21 patients.

### Cost analysis

The costs of each additional investigation were: US abdominal Doppler, $240.2 (*SD* = 16.3); US abdominal, $158.9 (*SD* = 12.4); CTAP, $652.0 (*SD* = 44.7); MRCP, $583.2 (*SD* = 46.1); ERCP, $4265.3 (*SD* = 1669.4); ultrasound-guided LBx, $634.2 (*SD* = 315.0); and T-tube cholangiogram, $108.4 (*SD* = 9.1).

Comparing the uncomplicated and complicated LFT groups, the mean costs of additional investigation were $2255.2 (*SD* = 2509.5) in the complicated LFT group and $391.8 (*SD* = 828.9) in the uncomplicated LFT group. Patients with complicated LFTs cost $1863.3 more to investigate than those with uncomplicated LFTs (95% CI [1289.0–2437.6], *p* < 0.001). The cost difference (per patient) between patients with uncomplicated and complicated LFTs, stratified by investigation modality, is shown in Table 1.

**Table 1:** Mean per-patient cost difference between complicated liver function test (LFT) versus uncomplicated LFT groups, stratified by investigation modality

Investigation modality	Difference between groups *Mean* $ (95% CI)	*p*
US doppler outside 4 days	92.6 (34.9–150.4)	0.003
CTAP	327.9 (174.7–481.0)	< 0.001
MRCP	128.4 (56.9–200.0)	< 0.001
ERCP	929.7 (416.9–1442.5)	< 0.001
LBx	315.4 (224.6–406.1)	< 0.001
Cholangiogram	63.3 (40.1–86.5)	< 0.001

*US* ultrasound, *CTAP* computed tomography abdomen pelvis, *MRCP* magnetic resonance cholangio-pancreatography, *ERCP* endoscopic retrograde cholangio-pancreatography, *LBx* liver biopsy

In patients with complicated LFT pattern, the mean cost of the additional investigations were $2710.8 (*SD* = 2344.2) in patients with biopsy-proven rejections, $2498.4 (*SD* = 3059.7) in patients with a biopsy not showing rejection, and $1845.4 (*SD* = 2469.5) in those without liver biopsy (p = 0.33). The mean cost of additional investigation prior to LBx in patients with a complicated LFT was $703.2 (*SD* = 955.9) with no statistical difference observed in the cost of additional investigation prior to LBx between those with biopsy-proven rejection ($795.8, *SD* = 1083.0) and those with a biopsy not showing rejection ($442.2, *SD* = 355.0, *p* = 0.12).

Besides additional investigations, patients with a complicated LFT also required longer intensive care (6.9 days cf. 4.2 days, *p* = 0.01) and a longer acute ward stays (25.8 days cf. 16.2 days, *p* < 0.001).

## Discussion

In this study, we quantified the number, type, and cost of additional investigations in patients undergoing LT. LFT is necessary for post-transplant monitoring. However, LFT is not sufficiently specific for distinguishing rejections from other complications. The study found that most patients with rejections, were not investigated with liver biopsy first, instead, they were investigated with other imaging modalities first. It remains difficult to determine which patient will benefit from an early LBx, using LFT alone. While there is no statistically significant difference in costs when stratified by biopsy results, the mean cost of additional investigation prior to LBx in patients with a complicated LFT was $703.2 (*SD* = 955.9). There is a need for more streamlined and evidence-based pathways for the investigations of LFT derangement post-transplant.

Several approaches to optimize post-transplant monitoring exist. In the past, aggressive protocol LBx at predefined timepoints irrespective of LFT has been proposed [5]. It has been argued that an LBx, whether driven by LFT derangement or not, can provide information additional to LFTs and imaging, which can help direct patient care [5]. However, the aggressive use of LBx to work up abnormal LFTs has not gained widespread acceptance due to its invasive nature and the risk of bleeding, infection, biliary leak, viscera injury and sampling error [5]. To balance the risks and benefits of LBx, most institutions, including ours, rarely perform LBx as an initial additional investigation for an abnormal LFT. Instead, we first perform less-invasive additional investigations, such as a US Doppler or cholangiogram, before a LBx. However, our study has demonstrated that this process can be further optimized. The key issue remains with patient selection—as an LFT is poorly specific, it is difficult to determine, without unnecessary imaging, which patient will benefit from an early LBx.

Another approach to streamline post-transplant monitoring is micro-analysis of LFT markers. However, it remains controversial if any one specific liver enzymes can be used to predict complications such as rejection, with a degree of specificity sufficiently high to guide diagnostic and therapeutic decision. Existing studies reported mixed and varied outcome on this approach. Most studies concluded that individual LFT markers although at times correlated with complication outcomes, it ultimately still lacks the required specificity to significantly alter post-transplant monitoring [1, 2, 6–10]. Consequently, there are no consensus society guidelines that propose investigation choices based solely on a specific LFT marker.

Recently, novel biomarkers such as donor cell free DNA (ddcfDNA) are being investigated in hope of streamlining post-transplant monitoring. A recent study, ddcfDNA had significantly better diagnostic performance than LFTs in detecting biopsy-proven rejection (98.8% versus ALT, 85.7%; ALP, 66.4%; GGT, 80.1%; and bilirubin 35.4%) [11]. Similar results have been reported in other international studies [12]. These early results showed that novel biomarkers may help discriminate between rejection-related and other pathology-related LFT derangement to guide investigations choices. However, novel biomarkers are still in their nascent phase of research and development. They cannot be used to distinguish between different LT complications without additional investigations. While it may have the potential to help choose which additional investigations, further prospective studies are still required to evaluate their diagnostic performances.

## Limitations

Our study is limited by its retrospective observational design and small patient numbers. As a single center study, our findings may also not be generalizable to other LT units in Australia and internationally. Finally, we acknowledge that this is a descriptive audit and that we have not investigated whether any of the biochemical test performed predict post-transplant graft dysfunction.

## Supplementary Information

**Additional file 1: Figure S1. A** is an example of an uncomplicated liver function test (LFT) profile post–liver transplant, while **B** is an example of a complicated LFT profile post–liver transplant. ALP: alkaline phosphatase; ALT: alanine aminotransferase; GGT: gamma-glutamyltransferase. **Figure S2. **Flow chart of patient inclusion and exclusion from the study. **Figure S3. **Comparison of the number of additional investigations in the uncomplicated versus complicated liver function test (LFT) groups. **Figure S4.** LFT profile and clinical characteristics of the six uncomplicated liver function test (LFT) patients with liver biopsies (LBxes). ALP: alkaline phosphatase; ALT: alanine aminotransferase; GGT: gamma-glutamyltransferase, LT: liver transplant; ETOH: ethanol; PSC: primary sclerosing cholangitis; NASH: non-alcoholic steatohepatitis; HCC: hepatocellular carcinoma; MRCP: magnetic resonance cholangio-pancreatography; ACR: acute cellular rejection; RTT: return to theatre; HCV: hepatitis C virus; HBV: hepatitis B virus. **Table S1. **Baseline characteristics of patients with uncomplicated liver function tests versus complicated LFTs. MELD: model for end-stage liver disease.

## Data Availability

Data available upon reasonable request via Dr. DZL.

## Abbreviations

LT: Liver transplantation
LFT: Liver function test
LBx: Liver biopsy
US: Ultrasound
CT: Computed tomography
CTAP: Computed tomography abdomen pelvis
MRI: Magnetic resonance imaging
ERCP: Endoscopic retrograde cholangio-pancreatography
MRCP: Magnetic retrograde cholangio-pancreatography (MRCP)
STROBE: Strengthening the reporting of observational studies in epidemiology
ALP: Alkaline phosphatase
ALT: Alanine aminotransferase
GGT: Gamma-glutamyltransferase
UCLA: University of california, los angeles
RAI: Rejection activity index
ddcfDNA: Donor cell free deoxyribonucleic acid
MELD: Model for end-stage liver disease
ETOH: Ethanol
PSC: Primary sclerosing cholangitis
NASH: Non-alcoholic steatohepatitis
HCC: Hepatocellular carcinoma
ACR: Acute cellular rejection
RTT: Return to theatre
HCV: Hepatitis C virus
HBV: Hepatitis B virus
CI: Confidence interval
SD: Standard deviation
