# Supplementary material for: Clinical and financial impacts of abnormal liver biochemistry after liver transplantation
Source: BMC Res Notes. 2023 Jan 27;16:7. doi: 10.1186/s13104-022-06268-w (PMC9883895; doi:10.1186/s13104-022-06268-w)
Supplement: Supplementary file 1 — Additional file 1: Figure S1. A is an example of an uncomplicated liver function test (LFT) profile post–liver transplant, while B is an example of a complicated LFT profile post–liver transplant. ALP: alkaline phosphatase; ALT: alanine aminotransferase; GGT: gamma-glutamyltransferase. Figure S2. Flow chart of patient inclusion and exclusion from the study. Figure S3. Comparison of the number of additional investigations in the uncomplicated versus complicated liver function test (LFT) groups. Figure S4. LFT profile and clinical characteristics of the six uncomplicated liver function test (LFT) patients with liver biopsies (LBxes). ALP: alkaline phosphatase; ALT: alanine aminotransferase; GGT: gamma-glutamyltransferase, LT: liver transplant; ETOH: ethanol; PSC: primary sclerosing cholangitis; NASH: non-alcoholic steatohepatitis; HCC: hepatocellular carcinoma; MRCP: magnetic resonance cholangio-pancreatography; ACR: acute cellular rejection; RTT: return to theatre; HCV: hepatitis C virus; HBV: hepatitis B virus. Table S1. Baseline characteristics of patients with uncomplicated liver function tests versus complicated LFTs. MELD: model for end-stage liver disease. [file 13104_2022_6268_MOESM1_ESM.docx]

**Supplementary Materials**


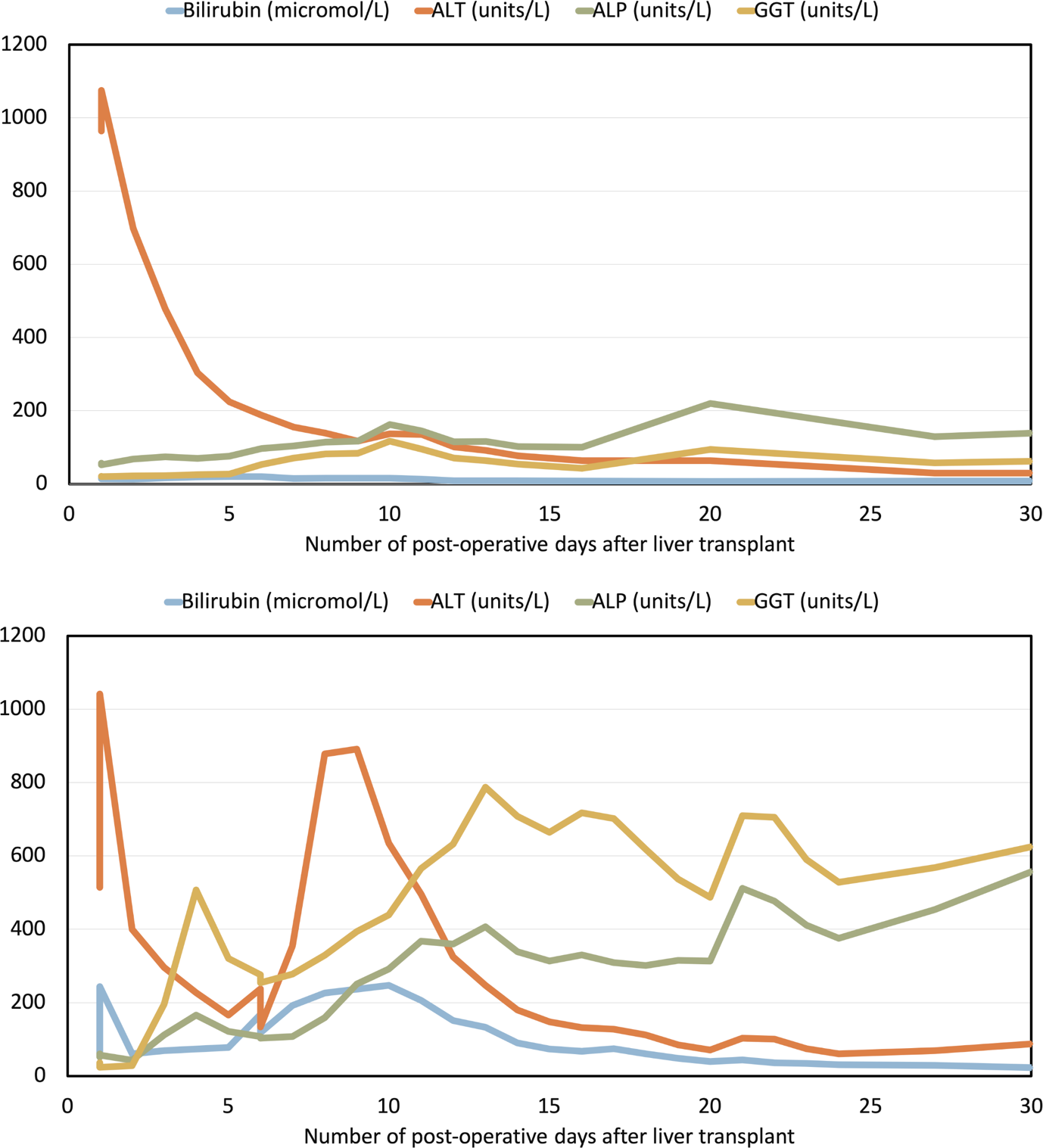
**Figure S1:** (A) is an example of an uncomplicated liver function test (LFT) profile post–liver transplant, while (B) is an example of a complicated LFT profile post–liver transplant. ALP: alkaline phosphatase; ALT: alanine aminotransferase; GGT: gamma-glutamyltransferase.

**
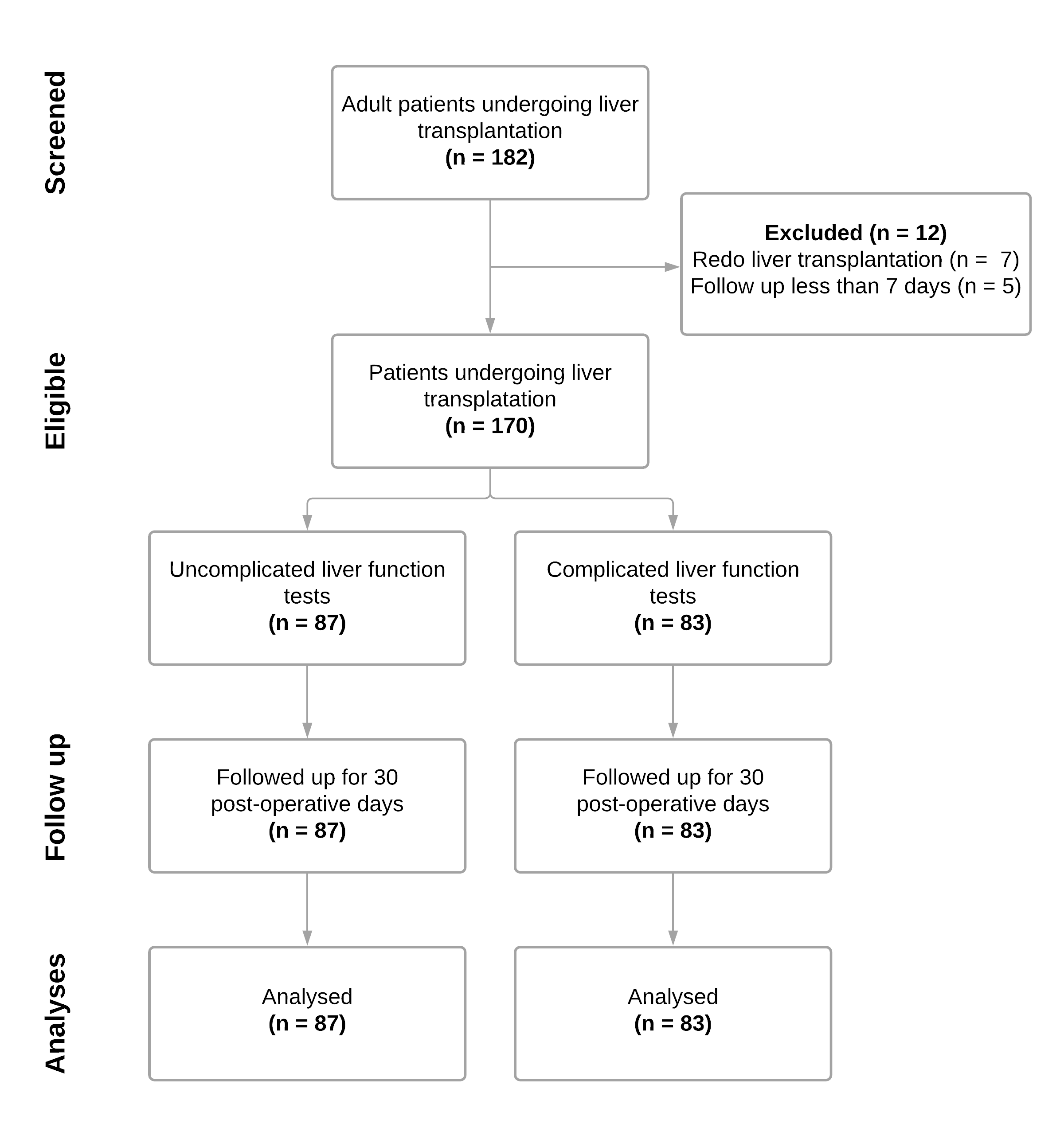
Figure S2:** Flow chart of patient inclusion and exclusion from the study.


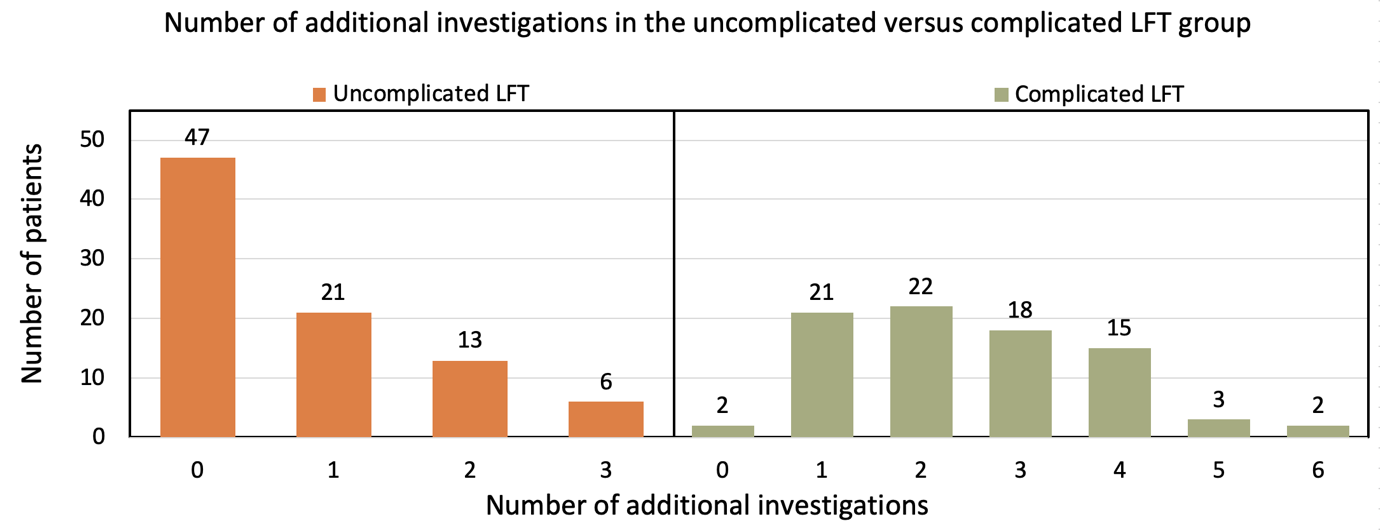


**Figure S3:** Comparison of the number of additional investigations in the uncomplicated versus complicated liver function test (LFT) group


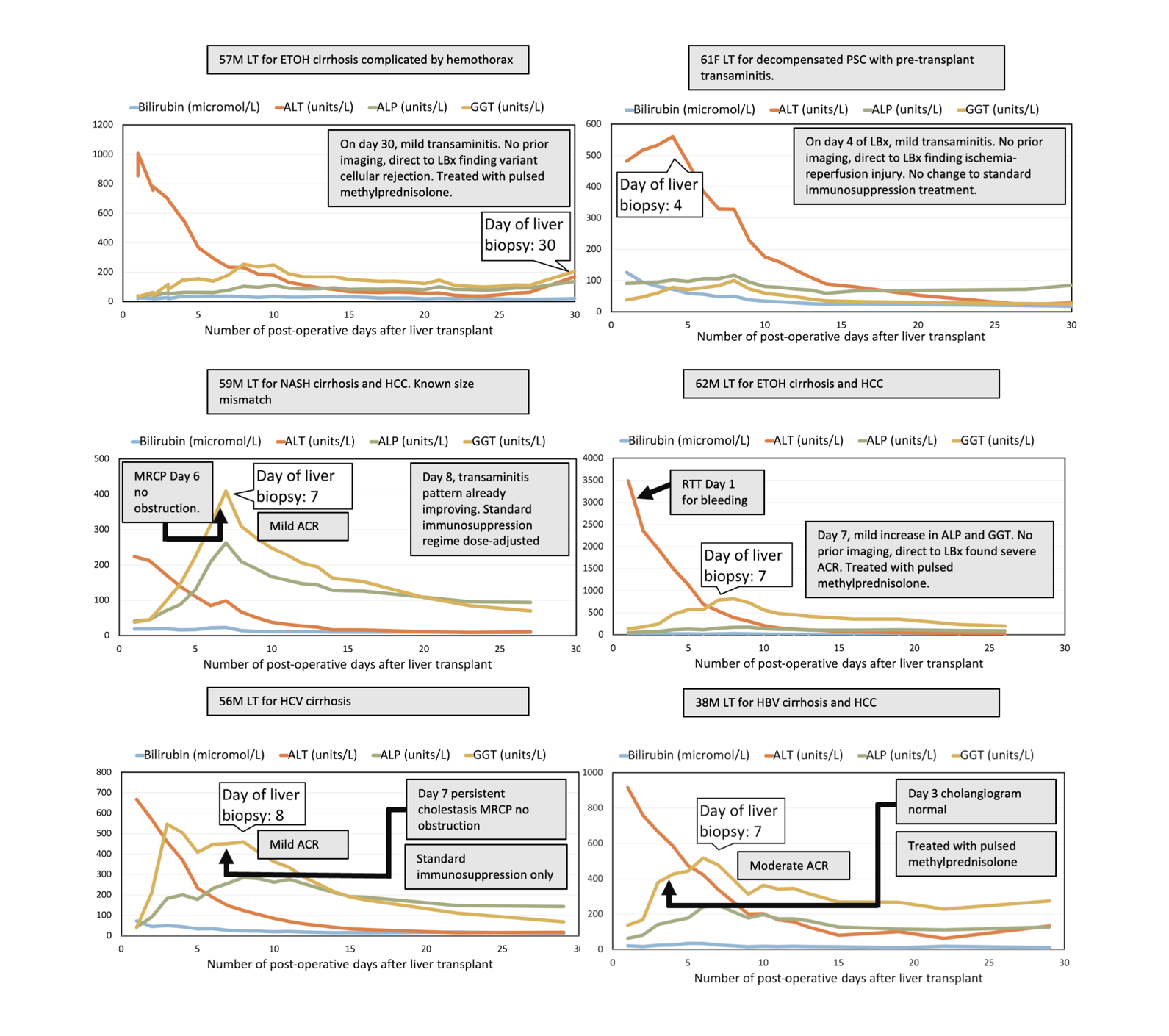
**Figure S4:** LFT profile and clinical characteristics of the six uncomplicated liver function test (LFT) patients with liver biopsies (LBxes). ALP: alkaline phosphatase; ALT: alanine aminotransferase; GGT: gamma-glutamyltransferase, LT: liver transplant; ETOH: ethanol; PSC: primary sclerosing cholangitis; NASH: non-alcoholic steatohepatitis; HCC: hepatocellular carcinoma; MRCP: magnetic resonance cholangio-pancreatography; ACR: acute cellular rejection; RTT: return to theatre; HCV: hepatitis C virus; HBV: hepatitis B virus.

**Table S1:** Baseline characteristics of patients with uncomplicated liver function tests versus complicated LFTs. MELD: model for end-stage liver disease.

|  | **LFT** | | ***p*** |
| --- | --- | --- | --- |
|  | **Uncomplicated**  **(*n* = 87)** | **Complicated**  **(*n* = 83)** |  |
| **Demographic variables** |  |  |  |
| Age, *M* (*SD*) | 55.0 (8.9) | 54.6 (10.8) | 0.79 |
| Sex, male, % | 70.1 | 61.4 | 0.23 |
| Body mass index, *M* (*SD*) | 28.5 (5.8) | 29.2 (6.7) | 0.44 |
| Child–Pugh score, *n* (%) |  |  | 0.36 |
| A | 16 (18.4) | 9 (10.8) |  |
| B | 22 (25.3) | 21 (25.3) |  |
| C | 49 (56.3) | 53 (63.9) |  |
| MELD score, *M* (*SD*) | 21.9 (9.7) | 23.8 (9.9) | 0.20 |
| **Operative variables (min.), *M* (*SD*)** |  |  |  |
| Warm ischemia | 47.7 (11.2) | 46.4 (9.7) | 0.41 |
| Cold ischemia | 397.7 (158.4) | 396.5 (104.2) | 0.95 |
| Operation time | 471.5 (103.1) | 488.0 (120.5) | 0.34 |
| **Indications for transplant, *n* (%)** |  |  |  |
| Liver cirrhosis | 39 (44.8) | 46 (55.4) | 0.17 |
| Hepatocellular carcinoma | 41 (47.1) | 29 (34.9) | 0.11 |
| Acute liver failure | 6 (6.9) | 5 (6.0) | 0.82 |
| Others | 1 (1.1) | 3 (3.6) | 0.36 |
| **Liver pathology, *n* (%)** |  |  |  |
| Primary sclerosing cholangitis | 11 (12.6) | 12 (14.5) | 0.73 |
| Primary biliary cirrhosis | 3 (3.4) | 3 (3.6) | 1.00 |
| Ethanol hepatitis | 15 (17.2) | 20 (24.1) | 0.27 |
| Non-alcoholic steatohepatitis | 10 (11.5) | 13 (15.7) | 0.43 |
| Hepatitis B | 13 (14.9) | 5 (6.0) | 0.06 |
| Hepatitis C | 28 (32.2) | 23 (27.7) | 0.53 |
| Metabolic syndrome | 2 (2.3) | 3 (3.6) | 0.68 |
| Autoimmune hepatitis | 4 (4.6) | 1 (1.2) | 0.37 |
| Others | 9 (10.3) | 10 (12.0) | 0.73 |
